# Tumor suppressor PRSS8 targets Sphk1/S1P/Stat3/Akt signaling in colorectal cancer

**DOI:** 10.18632/oncotarget.8511

**Published:** 2016-03-31

**Authors:** Yonghua Bao, Kai Li, Yongchen Guo, Qian Wang, Zexin Li, Yiqiong Yang, Zhiguo Chen, Jianguo Wang, Weixing Zhao, Huijuan Zhang, Jiwang Chen, Huali Dong, Kui Shen, Alan M. Diamond, Wancai Yang

**Affiliations:** ^1^ Department of Pathology and Institute of Precision Medicine, Jining Medical University, Jining 272067, China; ^2^ Department of Pathology, the First Affiliated Hospital, Xinxiang Medical University, Xinxiang 453003, China; ^3^ Department of Immunology, Xinxiang Medical University, Xinxiang 453003, China; ^4^ Department of Surgical Oncology, the First Affiliated Hospital, Xinxiang Medical University, Weihui 453003, China; ^5^ Department of Pathology, Xinxiang Medical University, Xinxiang 453003, China; ^6^ Division of Pulmonary, Critical Care, Sleep and Allergy, Department of Medicine, University of Illinois at Chicago, Chicago, IL 60612, USA; ^7^ Department of Pathology, University of Illinois at Chicago, Chicago, IL 60612, USA

**Keywords:** PRSS8, colorectal cancer, tumor suppressor, Sphk1, Stat3

## Abstract

PRSS8 is a membrane-anchored serine protease prostasin and has been shown an association with carcinogenesis. Herein we found that PRSS8 expression was significantly reduced in colorectal adenomas and adenocarcinomas. The decreased PRSS8 was well correlated with clinical stages, poor differentiation and shorter survival time of colorectal cancer. Furthermore, increase of PRSS8 led to the inhibition of colorectal cancer cell proliferation, knockdown of PRSS8 accelerated cell proliferation *in vitro*, and overexpressing PRSS8 retarded cancer cell growth in nude mice. Mechanistic studies revealed that PRSS8 inhibited Sphk1/S1P/Stat3/Akt signaling pathway, in terms of inverse association between PRSS8 and Sphk1 in human colorectal cancers and in *Sphk1-/−* mice. In conclusion, PRSS8 acts as a tumor suppressor by inhibiting Sphk1/S1P/Stat3/Akt signaling pathway, and could be used as a biomarker to monitor colorectal carcinogenesis and predict outcomes.

## INTRODUCTION

Colorectal cancer (CRC) is the third most common malignant disease and the second leading cause of cancer-related death in the United States [1], which is majorly due to cancer metastasis. Several decades effort have revealed that the development and progression of colorectal cancer is linked to the inactivation or downregulation of tumor suppressors, and activation or upregulation of oncogenes, leading to oncogenic signaling activation such as Wnt/β-catenin [2–4], inflammatory signaling [5, 6], etc. Using gene expression analysis we have found that PRSS8 was significantly reduced in colorectal cancer, and the reduction of PRSS8 expression was associated with poor differentiation and poor outcome, and lead to alterations in Sphk1/S1P/Stat3 signaling, the later has been reported to be associated with colitis-associated colorectal cancer [7, 8].

PRSS8 (protease serine 8), also known as Prostasin, is a trypsin-like serine peptidase [9–11]. PRSS8 was preliminarily found highly expressed in normal prostate gland and seminal fluid, and other studies have reported that PRSS8 is overexpressed in epithelial cells of various tissues and is involved in terminal epithelial differentiation. In addition, PRSS8 has the physiological and pathophysiological functions of regulating of sodium and fluid levels via proteolysis of the epithelial sodium channel, and shows important roles in the epidermal barrier function, skin phenotypes, and embryonic viability [10, 11]. More importantly, both oncologic and tumor suppressing roles of PRSS8 have been demonstrated in ovarian cancers [12, 13], for instance, one study showed that PRSS8 levels were increased in the serum of ovarian cancer patients [14], and another study showed that PRSS8 expression was decreased in chemoresistant ovarian cancer patients and chemoresistant cell line, and forced overexpression of prostasin in ovarian cancer cells greatly induced cells death [15]. Moreover, recent studies have also demonstrated tumor suppressive roles of PRSS8 on prostate, breast, bladder and gastric cancers, in which PRSS8 expression was reduced in prostate [16, 17], breast [18], bladder [19] and gastric cancers [20], due to the hypermethylation of PRSS8. While it has been studied extensively in other cancers, the effect of PRSS8 on colorectal cancer is not clear.

The present study employed tissue microarrays of colorectal, esophageal and liver cancers as well as prostate and breast cancers, to investigate the expression of PRSS8 and its clinical significance in these cancers. Gain- and loss-of-expression studies were used to determine the biological functions of PRSS8 and underlying molecular mechanism in culture cells and in nude mice. The results generated from these experiments strongly suggested that PRSS8 is a tumor suppressor in colorectal cancer, which is through inhibiting Sphk1/S1P/Stat3 signaling pathways.

## RESULTS

### PRSS8 mRNA levels were reduced in colorectal adenoma and adenocarcinoma

**Figure 1:**
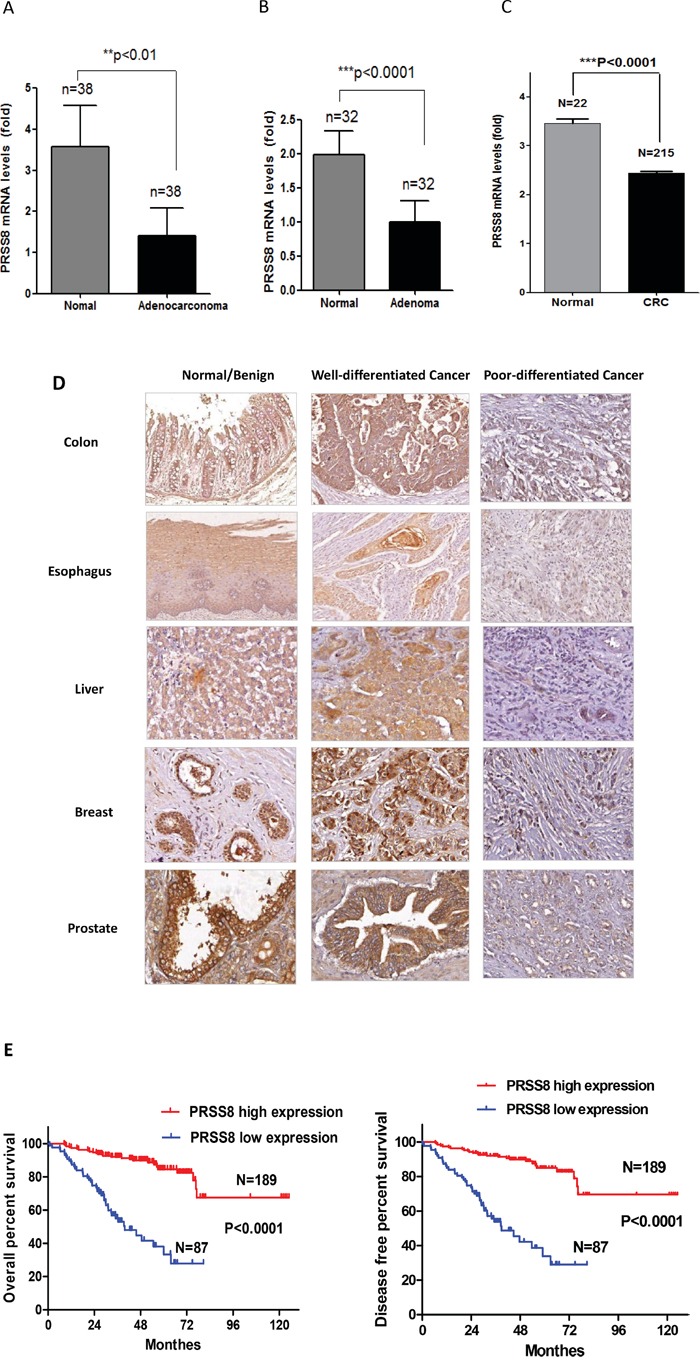
PRSS8 expression levels were reduced in tumors and the reduction of PRSS8 was associated with poor differentiation and survival time **A.** mRNA of PRSS8 (mean+/−SD) was reduced about 65% in colorectal cancer tissues with comparison to their paired non-cancer tissues. Total RNA was extracted from 38 paired frozen colorectal cancer/non-cancer tissues, and PRSS8 mRNA levels were analyzed and normalized to the corresponding levels of GAPDH, assayed by qRT-PCR. **B.** PRSS8 mRNA levels (mean+/−SD) were reduced about 50% in colorectal adenoma tissues with comparison to their paired non-tumor tissues. PRSS8 mRNA expression levels of colorectal adenoma were extracted from the online database (GEO Profile # GDS2947) (**p<0.01, ***p<0.001). **C.** The reduction of PRSS8 in colorectal cancer was also seen in The Cancer Genome Atlas (TCGA) online data (http://cancergenome.nih.gov/), in 215 cases of colorectal cancers, compared to 22 normal colorectal mucosa (p<0.0001). **D.** PRSS8 was reduced in colorectal cancers and the cancers of esophagus, liver, breast and prostate, compared to their normal (esophagus, colon and liver) or benign lesions (breast and prostate) assayed by immunohistochemical staining using anti-PRSS8 antibody. The reduced expression of PRSS8 was associated with poor differentiation. **E.** PRSS8 protein levels were associated with the survival time and disease-free period of the patients of colorectal cancers. The survival curve was generated using GraphPad Prism 5.0,.

To determine how PRSS8 expression differed between normal and tumor colorectal tissues, we used qRT-PCR to measure PRSS8 mRNA levels in 38 pairs of colorectal cancer tissues and their adjacent non-cancer tissues. As shown in Figure 1A, PRSS8 mRNA levels were reduced about 65% in adenocarcinoma tissues compared with non-cancer tissues (p<0.01). To determine whether loss of PRSS8 occurred in the early stage of tumor progression, we used an online gene expression data set to compare 32 paired patient adenoma tissues and their adjacent normal colonic mucosa (GEO# GDS2947/202525/PRSS8). Similar to adenocarcinoma, PRSS8 mRNA was reduced about 50% in adenoma compared to normal mucosa (p<0.0001, Figure 1B). The reduction of PRSS8 in colorectal cancer was also supported by mining The Cancer Genome Atlas (TCGA) online data (http://cancergenome.nih.gov/) [21], showing that PRSS8 was significantly reduced in 215 cases of colorectal cancers in comparison to the normal colorectal mucosa (Figure 1C, p<0.0001). These finding indicated that PRSS8 mRNA levels were significantly reduced during colorectal cancer progression.

### PRSS8 protein levels were reduced in colorectal cancers and the cancers of esophagus, liver, breast and prostate

To determine whether PRSS8 protein levels in colorectal cancers were also reduced, we conducted immunohistochemical staining in a colorectal cancer tissue microarray (TMA) that contained 282 cases of colorectal cancer with follow-up information. Compared to the adjacent non-cancer tissues, colorectal cancers showed significant reduction of PRSS8 expression (Figure 1D). Interestingly, the reduction of PRSS8 was positively associated with cancer differentiation. For instance, PRSS8 protein level was highly expressed in well differentiated colorectal adenocarcinomas and was lower or absent in poorly differentiated colorectal adenocarcinomas (Figure 1D, Table 1, p<0.00001).

**Table 1:** The association between PRSS8 expression and the clinicopathological characteristics

Clinicopathologic Variables	N	PRSS8 expression score			P value
		3	2	1	
**Age**					
Patient number (N=282)		119	93	70	
Median age (years)		59.0	56.5	62.0	0.822
Range		27-82	36-86	33-85	
**Gender**					0.088
Male	143	69 (48%)	40 (28%)	34 (24%)	
Female	139	50 (36%)	53 (38%)	36 (26%)	
**Lymphatic metastasis**					0.157
Yes	111	41 (37%)	36 (32%)	34 (31%)	
No	171	78 (46%)	57 (33%)	36 (21%)	
**Clinical stages**					**0.0046**
1-2	149	70 (47%)	50 (34%)	29 (19%)	
3-4	133	42 (32%)	44 (33%)	47 (35%)	
**Differentiation**					**<0.00001**
High	135	74 (55%)	37 (27%)	24 (18%)	
Moderate	112	40 (36%)	45 (40%)	27 (24%)	
Low	35	5 (14%)	12 (34%)	18 (51%)	

N, number of patients with colorectal cancers.

Previous studies have demonstrated that PRSS8 was differentially expressed in ovarian cancer [15], prostate cancer [16, 17] and breast cancer [18]. To determine the levels of PRSS8 in other cancers, we used 4 sets of TMAs that contained 72 pairs of esophageal cancers and their adjacent non-cancer tissues, 117 pairs of liver cancers and their adjacent non-cancer tissues, 46 pairs of prostate cancers and their adjacent adenoma or non-cancer tissues, and 75 pairs of breast cancers and their adjacent adenoma or non-cancer tissues, respectively. Similar to colorectal cancer, PRSS8 expression levels were reduced in esophageal cancers and liver cancers compared to normal adjacent tissues (Figure 1D). Consistent with previous studies, PRSS8 expression was reduced in breast and prostate cancers, compared to breast and prostate adenomas (Figure 1D). Moreover, PRSS8 expression was positively associated with differentiation of esophageal, liver, breast and prostate cancers (Figure 1D)

### PRSS8 expression was associated with survival of colorectal cancer patients

Since reduced expression of PRSS8 was seen in colorectal adenoma and adenocarcinoma (Figure 1D), we then determined whether PRSS8 expression was linked to clinicopatholgical characteristics and outcomes. As shown in Figure 1E, reduced PRSS8 was significantly associated with shorter survival time and disease-free period of patients with colorectal cancer (Figure 1E, p<0.0001). In addition, there was an inverse correlation between PRSS8 expression levels and colorectal cancer stages (Table 1, p=0.0046). Taken together, above findings strongly demonstrated the important clinical significance of PRSS8 in cancer formation, progression and clinical outcomes.

### PRSS8 affected colorectal cancer cell proliferation, cell cycle

**Figure 2:**
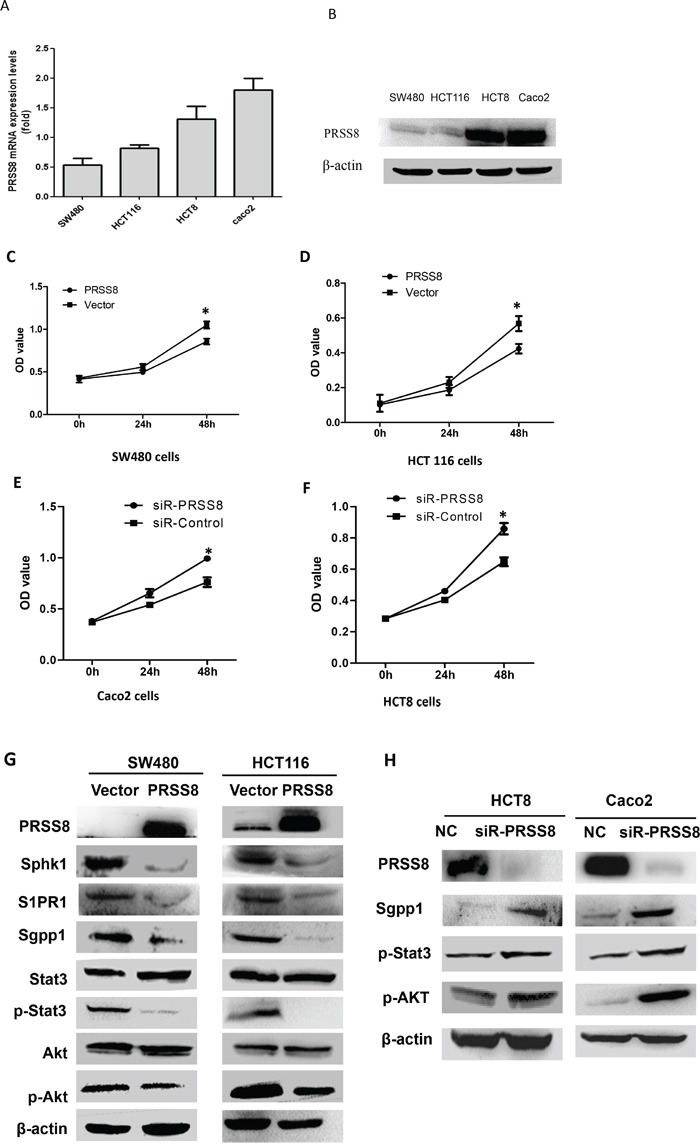
Differential expression and biological functions of PRSS8 in human colorectal cancer cell lines **A.** PRSS8 mRNA levels were analyzed by qRT-PCR and normalized to GAPDH. **B.** PRSS8 proteins were evaluated by immunoblotting, and β-actin was used as internal control. **C** and **D.** overexpressing PRSS8 inhibited cancer cell proliferation in SW480 (C) and HCT116 cells (D). **E** and **F.** knockdown PRSS8 by small interfering RNA accelerated cell proliferation in Caco2 (E) and HCT8 cells (F). **G.** increased expression of PRSS8 decreased the expression of Sphk1, S1P receptor (S1PR), Sgpp1, phosphorylated Stat3 (p-Stat3), and phosphorylated Akt (p-Akt) in both HCT116 and SW480 cell lines. **H.** knockdown of PRSS8 expression led to increase of Sgpp1, p-Stat3 and p-Akt in HCT8 and Caco2 cell lines, assayed by immunoblotting.

Due to the strong correlation between PRSS8 expression levels and clinical significance, we then used gain- and loss-of-expression approaches to determine the biological functions of PRSS8 in colorectal cancer *in vitro* and in nude mice. Four human colorectal cancer cell lines were screened for PRSS8 mRNA and protein levels by qRT-PCR and immunoblotting, respectively. PRSS8 mRNA levels (Figure 2A) and protein levels (Figure 2B) were lower in SW480 and HCT116 cells and higher in HCT8 and Caco2 cells. SW480 and HCT116 cells, that have lower expression of PRSS8, were chosen for transfection with PRSS8 expression plasmid (pFlag-PRSS8). MTT assay showed that increasing PRSS8 expression inhibited cancer cell proliferation at 24 and 48 hours in both SW480 and HCT116 cell lines (Figure 2C, 2D, p<0.05), compared to the cells transfected with empty vector.

We then determined whether PRSS8 expression was correlated with the cell cycle. Flow cytometry analysis showed that increased expression of PRSS8 led to cell cycle arrest at G1/G2 phase at both SW480 and HCT116 cells (Table 2, p<0.05, compared to the cells transfected with empty vector).

**Table 2:** Overexpression of PRSS8 caused cell cycle arrest at G1/G2 phases in human colorectal cancer cells

	SW480		HCT116	
	Vector	PRSS8	Vector	PRSS8
**G1 (%)**	36.2	53.5*	34.9	53.7*
**G2 (%)**	25.7	31.4*	7.4	30.9*
**S (%)**	38.1	15.1	57.7	15.4
**Total (%)**	100.0	100.0	100.0	100.0

**Note:** Human colorectal cancer cells SW480 and HCT116 were transfected with PRSS8 expression plasmids. 48 hours after transfection, the cells were collected for cell cycle analysis. The results showed that increased expression of PRSS8 caused cell cycle arrest in G1/G2 phases, compared to the cells with transfection of empty vector, assayed by flow cytometry. (*p<0.05). PRSS8, the cells with transfection of PRSS8 expression plasmid pFlag-PRSS8; Vector, the cells with transfection of empty vector.

To determine the effects of knockdown of PRSS8 expression in colorectal cancer cells, we decreased expression of PRSS8 of the high-expression colorectal cancer cell lines Caco2 and HCT8 using small interfering RNA (siRNA) targeting human PRSS8 (siR-PRSS8). MTT results showed that knockdown of PRSS8 significantly promoted cell proliferation (Figure 2E and 2F).

### PRSS8 suppressed Sphk1/S1P/Stat3/Akt signaling

It has been known that malignant transformation of chronic colitis is another major cause of colorectal cancer formation and progression besides the activation of Wnt-β-catenin signaling. A recent study has reported that persistent activation of Stat3 by the Sphk1/S1P signaling pathway is observed in chronic intestinal inflammation and colitis-associated colorectal cancer [8]. Thus, we determined whether PRSS8 was involved in the Sphk1/S1p/Stat3 signaling. Indeed, increased expression of PRSS8 remarkably decreased the expression of Sphk1, S1P receptor (S1PR), Sgpp1, phosphorylated Stat3 (p-Stat3), and phosphorylated Akt (p-Akt) in both HCT116 and SW480 cell lines (Figure 2G), although total of Stat3 and total Akt were not changed. In contrast, knockdown of PRSS8 upregulated the expression of Sgpp1, p-Stat3 and p-Akt in HCT8 and Caco2 cell lines (Figure 2H).

### The inverse correlation between PRSS8 and Sphk1

**Figure 3:**
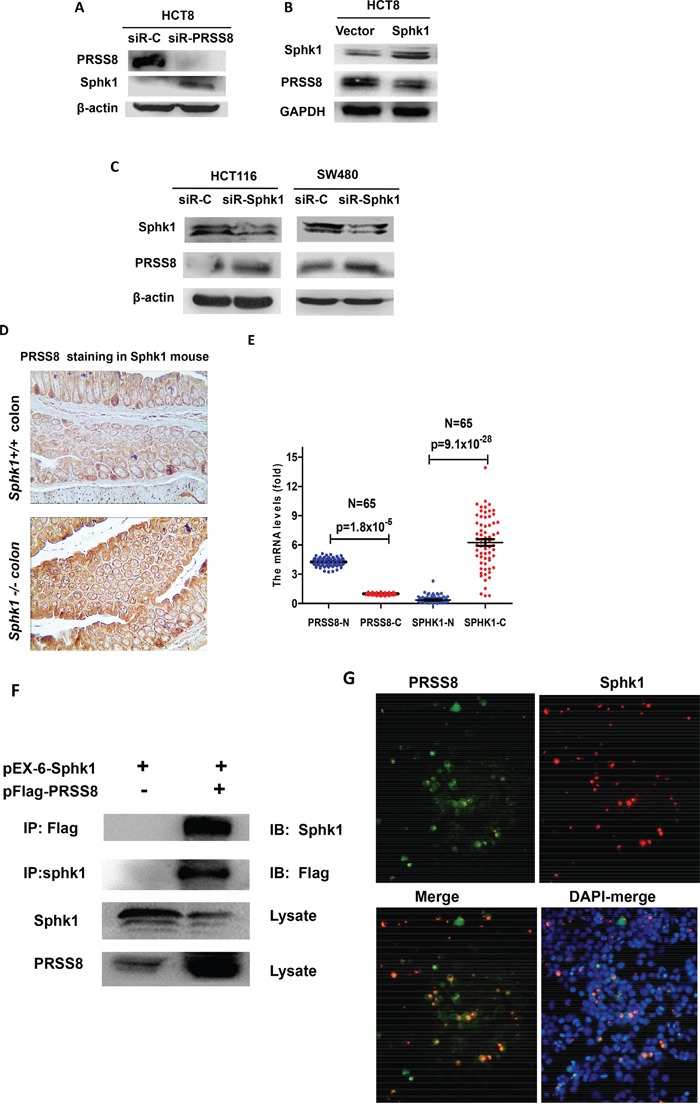
Negative interaction between PRSS8 and Sphk1/S1P/Stat3/Akt signaling in colorectal cancer cells, in Sphk1 mouse models and in human colorectal cancers **A.** knockdown of PRSS8 led to the upregulation of Sphk1 in HCT8 cells. **B.** overexpression of Sphk1 in HCT8 cells suppressed PRSS8 expression. **C.** knockdown of Sphk1 expression caused upregulation of PRSS8 in HCT116 and SW480 cells. **D.** PRSS8 expression was increased in Sphk1-/− mouse colon (lower panel), compared to the Sphk1+/+ mice (upper panel). **E.** Online gene profile data set (www.oncomine.com) was deeply mined and neutralized, the results showed that PRSS8 was higher and Sphk1 was lower in normal colorectal mucosa, but RPSS8 was reduced (p=1.8×10^−5^) and Sphk1 was increased in colorectal cancers (p=9.1×10^−28^, Figure 3E), exhibiting a strong negative correlation. **F.** the interaction between PRSS8 and Sphk1 was examined by co-immunoprecipitation assay. PRSS8/Sphk1 complex was precipitated using anti-Flag or anti-Sphk1 antibodies, probed with anti-Sphk1 or anti-Flag antibodies, respectively. **G.** The interaction between PRSS8 and Sphk1 was also confirmed by immuno-fluorescence staining assay, showing that PRSS8 protein (p-EGFP-PRSS8, Green-fluorescence staining) was co-localized with Sphk1 protein (pEx-6-Sphk1, Red-fluorescence staining) in cytoplasm and nuclei.

Interestingly, knockdown of PRSS8 could cause Sphk1 upregulation in HCT8 cells (Figure 3A). Moreover, increasing expression of Sphk1 in HCT8 cells led to the downregulation of PRSS8 (Figure 3B), and vice versa, knockdown of Sphk1 by small interfering RNA targeting Sphk1 in HCT116 and SW480 cells led to the upregulation of PRSS8 (Figure 3C).

The negative regulation between PRSS8 and Sphk1 was also observed in Sphk1 mouse model. As shown in Figure 3D, PRSS8 expression was increased in the colon of *Sphk1-/−* mice, compared to *Sphk1+/+* mice, assayed by immunohistochemical staining.

The negative association between PRSS8 and Sphk1 was evidenced by a published gene profile data set (www.oncomine.com)[22]. The data was deeply mined and results showed that in the 65 pairs of colorectal normal and cancers, PRSS8 was higher and Sphk1 was lower in normal colorectal mucosa, but RPSS8 was significantly reduced (p=1.8×10^−5^) and Sphk1 was dramatically increased in colorectal cancers (p=9.1×10^−28^, Figure 3E), exhibiting a strong negative correlation.

The aforementioned gain- and loss-of-expression studies of PRSS8 and Sphk1 showed a negative regulation of PRSS8 and Sphk1/ S1P/Stat3 signaling *in vitro* and *in vivo*, leading us to determine whether there is a direct interaction between PRSS8 and Sphk1. Using co-immunoprecipitation (co-IP) assay, we co-transfected the HEK 293 with PRSS8 expression plasmid (pFlag-PRSS8) and Sphk1 expression plasmid (pEx-6-Sphk1), and then precipitated the PRSS8/Sphk1 complex with anti-Flag or anti-Sphk1 antibodies, and immunoblotted with anti-Sphk1 or anti-Flag antibodies, respectively. We found that there was a strong band between PRSS8 and Sphk1 (Figure 3F, upper 2 bands). Notably, the Figure 3F lower 2 bands showed again an inverse correlation between Sphk1 and PRSS8, in which Sphk1 suppressed PRSS8 expression (in the whole lysate) and PRSS8 suppressed Sphk1 expression (in the whole lysate). The interaction between PRSS8 and Sphk1 was further confirmed by immuno-fluorescence staining assay (Figure 3G), which showed that PRSS8 protein (p-EGFP-PRSS8, Green-fluorescence staining) was co-localized with Sphk1 protein (pEx-6-Sphk1, Red-fluorescence staining) in the cytoplasm and nucleus.

### PRSS8 inhibited tumor growth in nude mice

**Figure 4:**
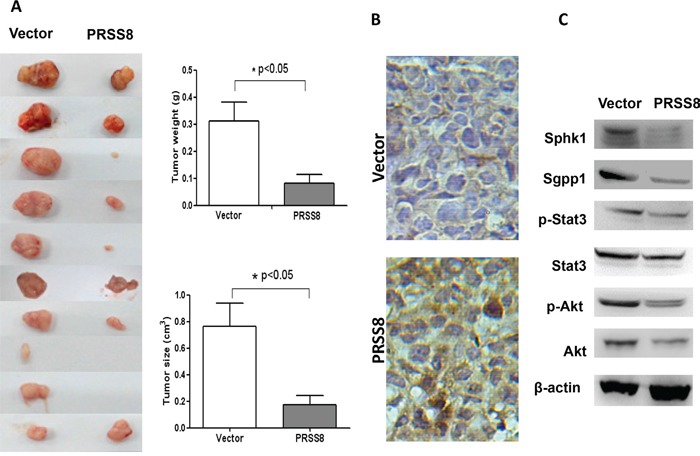
PRSS8 inhibited colon cancer cell growth in nude mice **A.** 1.5×10^6^ stable cell lines with transfection of empty vector (Vector) or PRSS8 expression plasmid (PRSS8) were injected subcutaneously in nude mice, and the mice were sacrificed 3 weeks post injection. PRSS8 inhibited tumors (xenografts) weight and size. **B.** expression levels of PRSS8 were validated in the tumors by immunohistochemical staining using anti-PRSS8 antibody. **C.** PRSS8 overexpression resulted in the downregulation of Sphk1/S1P/Stat3/Akt in nude mouse tumors.

To determine tumor suppressive roles of PRSS8 *in vivo*, we established a stable PRSS8 overexpression HCT116 cell line, and injected these cells subcutaneously in nude mice. As shown in Figure 4A, overexpressing PRSS8 significantly inhibited tumor growth in mice, resulting in significant retardation of tumor size and tumor weight. To further confirm whether tumor growth inhibition was directly resulted from PRSS8, PRSS8 protein levels were assayed by immunohistochemical staining. Results showed that PRSS8 was overexpressed in HCT116 tumor cells which were transfected with PRSS8 plasmid, and that PRSS8 was almost undetectable in the tumor cells that were transfected with empty vector (Figure 4B).

Consistent with the effects of PRSS8 on oncogenic signaling pathways *in vitro*, in the HCT116 cell xenografts PRSS8 also suppressed Sphk1/S1P/Stat3/Akt signaling (Figure 4C).

## DISCUSSION

Recent studies have made significant advances in understanding colorectal carcinogenesis, demonstrating the critical roles of non-canonical Wnt signaling in colorectal cancer formation and progression, and identifying the link between chronic colitis and colorectal cancer, particularly in deciphering the roles of cytokines and their downstream transcription factors NF-kB and STAT3 [3, 5, 7, 23, 24]. These studies have also demonstrated that a chronic inflammatory microenvironment accelerates Apc mutation-initiated intestinal tumor formation in the Apc/Muc2 double mutant mouse model of intestinal cancer [25], showing a synergistic role of Apc/Wnt/β-catenin and cytokine/Cox2 inflammatory signaling in colorectal carcinogenesis. However, it is unknown whether there are any upstream regulators that exhibit tumor suppressing or oncogenic roles in colorectal cancer progression and metastasis through suppressing of inflammatory signaling pathways. In the present study, we identified that PRSS8 expression was reduced in colorectal cancer and this reduced expression was associated with poor differentiation and late stages of tumors, and with poor outcome of colorectal cancer patients. Overexpressing PRSS8 inhibited cancer cell proliferation, led to cell cycle arrest at G1/G2 phase, and retarded cancer cell growth in nude mice. Mechanistic studies showed that PRSS8 inhibited Sphk1/S1P/Stat3 signaling.

Reduced expression of tumor suppressor genes by inactivation or silencing is frequently observed in late stage of colorectal cancer [26, 27], and it may be a result of tumor progression, not a driver. In this study, using five sets of variant TMAs, including colorectal, esophageal, liver, prostate, and breast cancers, we have found that PRSS8 expression was reduced in all of the five types of cancer tissues, and poorly differentiated cancers exhibited less or absent expression of PRSS8. Notably, the expression of PRSS8 was reduced in colorectal adenoma, a pre-cancer lesion. Most importantly, the reduced expression of PRSS8 was associated with shorter survival time in patients with colorectal cancers. *In vivo* studies have further demonstrated a tumor suppressing role of PRSS8, including inhibition of tumor growth in nude mice and cancer cell proliferation, and cell cycle arrest. It is strongly suggested that PRSS8 is a tumor suppressor and has important clinical significance in colorectal cancer.

Sphingosine-1-phosphate (S1P) is a pleiotropic bioactive sphingolipid metabolite produced by sphingosine kinase 1 (Sphk1) and regulates numerous cellular processes important for cell growth, survival, invasion, lymphocyte trafficking, vascular integrity, and the production of cytokines and chemokines [8, 28, 29]. Growing evidence has demonstrated that S1P is linked to chronic intestinal inflammation and colitis-associated cancer, in which upregulation of Sphk1 is needed. Recent studies have demonstrated that the Sphk1/S1P axis causes the activation of NF-kB and transcription factor STAT3 and connects chronic inflammation and colitis-associated colorectal cancer, providing a novel mechanism of colitis malignant transformation [8, 30]. We found that PRSS8 inhibited Sphk1/S1P/Stat3 signaling, in terms of negative correlation of PRSS8 and Sphk1 *in vitro*, in Sphk1 mouse model and in human colorectal cancers. Interestingly, in normal colonic mucosa, PRSS8 expression was higher and Sphk1 was lower, but, in colorectal cancer tissues, PRSS8 expression was decreased and Sphk1 expression was increased, showing an inverse correlation (Figure 3E). The decrease of PRSS8 during carcinogenesis could be resulted from promoter hypermethylation, and the increase of Sphk1 could be activated by upstream signaling (e.g. inflammatory signaling) during colorectal cancer initiation and progression. Moreover, PRSS8 physically bond with Sphk1, evidenced by co-immunoprecipitation assay and co-localized expression in cytoplasm and nucleus. These findings again show tumor inhibition of PRSS8 via its crosstalk with Sphk1/S1p/Stat3 signaling.

In conclusion, we have identified PRSS8 as a tumor suppressor in colorectal cancer formation and progression, and PRSS8 might be one of the key elements that suppress Sphk1/S1p/Stat3/Akt inflammatory signaling during colorectal carcinogenesis. PRSS8 could be a useful biomarker for monitoring colorectal carcinogenesis and progression and for predicting outcomes.

## MATERIALS AND METHODS

### Human cancer samples and tissue microarray (TMA)

Human colorectal and esophageal tissues were obtained from the Tissue Bank of the Laboratory for Cancer Signaling Transduction at Xinxiang Medical University, and from the Institute of Precision Medicine of Jining Medical University, China. The human esophageal cancer tissue microarray (TMA) and colorectal cancer TMA with survival information was made in our laboratory, and the human breast cancer TMA were purchased from Biomax Inc. (Rockville, MD). The human prostate cancer TMA was purchased from Cooperative Human Tissue Network (CHTN) (Columbus, OH), and the liver cancer TMA was purchased from the Cybrdi Inc. (Potomac, MD). All procedures were approved by the Institutional Review Board of Xinxiang Medical University and Jining Medical University, China.

### Immunohistochemical staining, staining intensity evaluation and survival analysis

Paraffin sections were treated with 3% H_2_O_2_, and then incubated with primary antibodies and biotinylated secondary antibodies. The immune complexes were visualized using the Strept Avidin-Biotin Complex kit (Boster Biological Tech. LTD., Wuhan, China). All stained slides were imaged using Aperio ImageScope software (version 11) for immunostaining intensity evaluation. The immunohistochemical staining was scored by three independent researchers, who were blinded to the clinical or pathological information of these patients. A semi-quantitative scale from 0 to 100% was used to grade the percentage of PRSS8-stained epithelial cells relative to all epithelial cells in each tissue core on the TMA slides. For convenient statistic analysis, the percentage scores were transferred as following scored: 0 (<5%), 1 (5–25%), 2 (25–50%) and 3 (>50 staining). Cancer patient survival analysis was performed using Kaplan-Meier method and GraphPad Prism 5.0 software (La Jolla, CA).

### Quantitative reverse-transcriptional polymerase chain reaction (qRT-PCR)

Total RNA was extracted from the frozen colorectal cancer and normal tissues using Trizol reagent (Invitrogen, Carlsbad, CA) following the manufacturer's protocol. qRT-PCR (Applied Biosystem Inc.) was used for mRNA quantification analysis. The primers for mRNA analysis for qRT-PCR are listed in the Supplementary Table 1.

### Cell culture

Human colorectal cancer cell lines HCT116, SW480, HCT8 and Caco2, and human embryonic kidney cells HEK293 from American Type Culture Collection (ATCC) (Manassas, VA), were reserved by Tumor Center of Cancer Hospital, Chinese Academy of Medical Sciences (Beijing, China) for pure research in Jan, 2015. The PCR-based STR analysis was used to characterize these cells by using *GenePrint*® 10 System Kit (Cat.# B9510) in Jun, 2015. The system contains all materials necessary to amplify STR regions of human genomic DNA, including a hot-start thermostable DNA polymerase. An internal lane standard (ILS) and allelic ladder are genotyping of amplified fragments, and the 2800M Control DNA is supplied as a positive control. The cells were maintained in a complete MEM medium. All cells were free of mycoplasma contamination. All media were supplemented with 10% FBS and antibiotics (10,000 U/ml penicillin, 10 μg/ml streptomycin). Cells were cultured at 37°C in a humidified atmosphere containing 5% CO_2_.

### Expression plasmids construction, small interfering RNA (siRNA) synthesis and transfection

The full length of PRSS8 and Sphk1 were cloned from human cDNA and inserted into pEGFP vector (Promega, Madison, WI), pFlag and pEX-6 vector (Shanghai GenePharma, Shanghai, China), to generate pEGFP-PRSS8, pFlag-PRSS8 and pEX-6-Sphk1 expression constructs, respectively. Three 19-nt siRNA oligonucleotides with 3′-dt extensions against PRSS8 and Sphk1 transcripts, and one scrambled siRNA (negative control) were designed, as shown in Supplementary Table 1. siRNAs were synthesized by Shanghai GenePharma Inc. (Shanghai, China). Twenty-four hours before transfection, 1.0×10^5^ cells were seeded in a 6-well plate. 4 μg of PRSS8 or Sphk1 expression plasmid or negative control plasmid was transfected into cells, using Lipofectamine 3000 (Invitrogen, Carlsbad, CA) following the manufacture's protocol.

### Co-IP and co-localization analysis

The correlation between PRSS8 and Sphk1 was determined by co-immunoprecipitation (co-IP) and co-localization analysis. For co-immunoprecipitation analysis, HEK 293 cells were plated in 10-cm dishes. The cells in Dish 1 and 2 were co-transfected with pFlag-PRSS8 and pEx-6-Sphk1 plasmids. The cells in Dish 3 and 4 were co-transfected with negative control and pEx-6-Sphk1 plasmids. After 48 h, the cells were washed twice with ice-cold phosphate-buffered saline (PBS) and then incubated on ice for 15 min in RIPA lysis buffer supplemented with protease inhibitor cocktail. Total cell lysate was centrifuged at 12 000 r.p.m. for 15 min at 4°C. 300 μg of lysate were incubated with 1μg the anti-Flag antibody (Tianjin Sungene Biotech Co., Ltd) for Dish 1, anti-Sphk1 antibody (Abcam) for Dish 2, anti-Flag antibody for Dish 3 and anti-Sphk1 antibody for Dish 4, for 1 h at 4°C, followed by addition of 20μl agarose beads (Santa Cruz Biotechnology) for overnight at 4°C. Agarose beads were washed five times in RIPA lysis buffer supplemented with protease inhibitor cocktail. Complexes were released from the agarose beads by boiling for 5 min in 2× gel electrophoresis loading buffer. The immunocomplex was separated on 12% sodium dodecyl sulfate-polyacrylamide gel electrophoresis, and immunoblotting was used to detect PRSS8 with anti-flag and to detect Sphk1 with anti-Sphk1 antibody, respectively.

For co-localization analysis, the cells grown on a chamber slide (BD Biosciences, San Jose, CA) were co-transfected with pEGFP-PRSS8 and pEX-6-Sphk1 plasmids. Twenty-four hours after transfection, the cells were fixed with 4% paraformaldehyde in PBS for 30 min and permeabilized by further treatment with 0.2% Triton X-100 for 10 min, followed by DAPI (4′,6-Diamidine-2′-phenylindole dihydrochloride) staining for 15min. The images were taken using confocal microscopy (Olympus, China).

### Cancer cell transplantation (xenograft) in nude mice

1.5×10^6^ HCT116 stably transfected with pFlag-PRSS8 or negative control plasmid were injected subcutaneously into the flank of the normal nude mice at age about 8 weeks (5 mice per group). The animals were maintained in a pathogen-free barrier facility and closely monitored by animal facility staff. 30 days after inoculation, the animals were sacrificed and the xenografts were isolated and observed, the weight (g) and size (cm^3^) of the xenografts were determined. In addition, half of the xenograft tumors were fixed in 10% buffered formalin, embedded in paraffin and sectioned for hematoxylin and eosin staining and for immunohistochemical staining for PRSS8 expression validation. The another half of the xenograft tumors were frozen in liquid nitrogen for mRNA and protein analysis by qRT-PCR and immunoblotting. All procedures were conducted according to the Animal Care and Use guideline approved by Animal Care Committee of Xinxiang Medical University and Jining Medical University, China.

### Sphk1+/+ and -/− mice

Sphk1-deficient mice (*Sphk1-/−*) and wild-type (*Sphk1+/+*) sibling mice were generated by mating Sphk1+/− mice (in C57BL/6 background), as described recently [31]. The *Sphk1+/+ and -/−* mice were sacrificed at about 8 weeks, and colon tissues were collected and fixed in formalin for immunohistochemical staining using anti-PRSS8 antibody. All immunohistochemical staining slides were imaged using Aperio ImageScope software (version 11) for immunostaining intensity evaluation. All procedures were conducted according to the Animal Care and Use guideline approved by University of Illinois at Chicago Animal Care Committee.

## SUPPLEMENTARY TABLES



## Abbreviations

PRSS8: protease serine 8
Stat3: signal transducer and activator of transcription 3
Sphk1: sphingosine kinase 1
S1PR: Sphingosine-1- Phosphate receptor

## References

[1] Siegel R, Naishadham D, Jemal A (2012). Cancer statistics, 2012. CA Cancer J Clin.

[2] Herbst A, Kolligs FT (2007). Wnt signaling as a therapeutic target for cancer. Methods Mol Biol.

[3] Buchanan FG, DuBois RN (2006). Connecting COX-2 and Wnt in cancer. Cancer Cell.

[4] Jass JR (2006). Colorectal cancer: a multipathway disease. Crit Rev Oncog.

[5] Grivennikov SI (2013). Inflammation and colorectal cancer: colitis-associated neoplasia. Semin Immunopathol.

[6] Saleh M, Trinchieri G (2011). Innate immune mechanisms of colitis and colitis-associated colorectal cancer. Nat Rev Immunol.

[7] Grivennikov SI, Karin M (2010). Dangerous liaisons: STAT3 and NF-kappaB collaboration and crosstalk in cancer. Cytokine Growth Factor Rev.

[8] Liang J, Nagahashi M, Kim EY, Harikumar KB, Yamada A, Huang WC, Hait NC, Allegood JC, Price MM, Avni D, Takabe K, Kordula T, Milstien S, Spiegel S (2013). Sphingosine-1-phosphate links persistent STAT3 activation, chronic intestinal inflammation, and development of colitis-associated cancer. Cancer Cell.

[9] Yu JX, Chao L, Chao J (1994). Prostasin is a novel human serine proteinase from seminal fluid. Purification, tissue distribution, and localization in prostate gland. J Biol Chem.

[10] Hooper JD, Bowen N, Marshall H, Cullen LM, Sood R, Daniels R, Stuttgen MA, Normyle JF, Higgs DR, Kastner DL, Ogbourne SM, Pera MF, Jazwinska EC, Antalis TM (2000). Localization, expression and genomic structure of the gene encoding the human serine protease testisin. Biochim Biophys Acta.

[11] Yu JX, Chao L, Ward DC, Chao J (1996). Structure and chromosomal localization of the human prostasin (PRSS8) gene. Genomics.

[12] Lopez-Otin C, Matrisian LM (2007). Emerging roles of proteases in tumour suppression. Nature reviews.

[13] Sarojini S, Tamir A, Lim H, Li S, Zhang S, Goy A, Pecora A, Suh KS (2012). Early detection biomarkers for ovarian cancer. J Oncol.

[14] Mok SC, Chao J, Skates S, Wong K, Yiu GK, Muto MG, Berkowitz RS, Cramer DW (2001). Prostasin, a potential serum marker for ovarian cancer: identification through microarray technology. J Natl Cancer Inst.

[15] Yan BX, Ma JX, Zhang J, Guo Y, Mueller MD, Remick SC, Yu JJ (2014). Prostasin may contribute to chemoresistance, repress cancer cells in ovarian cancer, and is involved in the signaling pathways of CASP/PAK2-p34/actin. Cell Death Dis.

[16] Chen LM, Zhang X, Chai KX (2004). Regulation of prostasin expression and function in the prostate. Prostate.

[17] Takahashi S, Suzuki S, Inaguma S, Ikeda Y, Cho YM, Hayashi N, Inoue T, Sugimura Y, Nishiyama N, Fujita T, Chao J, Ushijima T, Shirai T (2003). Down-regulated expression of prostasin in high-grade or hormone-refractory human prostate cancers. Prostate.

[18] Chen LM, Chai KX (2002). Prostasin serine protease inhibits breast cancer invasiveness and is transcriptionally regulated by promoter DNA methylation. International journal of cancer.

[19] Chen LM, Verity NJ, Chai KX (2009). Loss of prostasin (PRSS8) in human bladder transitional cell carcinoma cell lines is associated with epithelial-mesenchymal transition (EMT). BMC Cancer.

[20] Sakashita K, Mimori K, Tanaka F, Tahara K, Inoue H, Sawada T, Ohira M, Hirakawa K, Mori M (2008). Clinical significance of low expression of Prostasin mRNA in human gastric cancer. J Surg Oncol.

[21] TCGA (2012). Comprehensive molecular characterization of human colon and rectal cancer. Nature.

[22] Gaedcke J, Grade M, Jung K, Camps J, Jo P, Emons G, Gehoff A, Sax U, Schirmer M, Becker H, Beissbarth T, Ried T, Ghadimi BM (2010). Mutated KRAS results in overexpression of DUSP4, a MAP-kinase phosphatase, and SMYD3, a histone methyltransferase, in rectal carcinomas. Genes, chromosomes & cancer.

[23] Grivennikov SI, Greten FR, Karin M (2010). Immunity, inflammation, and cancer. Cell.

[24] Ben-Neriah Y, Karin M (2012). Inflammation meets cancer, with NF-kappaB as the matchmaker. Nat Immunol.

[25] Yang K, Popova NV, Yang WC, Lozonschi I, Tadesse S, Kent S, Bancroft L, Matise I, Cormier RT, Scherer SJ, Edelmann W, Lipkin M, Augenlicht L, Velcich A (2008). Interaction of Muc2 and Apc on Wnt signaling and in intestinal tumorigenesis: potential role of chronic inflammation. Cancer Res.

[26] Vogelstein B, Kinzler KW (2004). Cancer genes and the pathways they control. Nat Med.

[27] Vogelstein B, Papadopoulos N, Velculescu VE, Zhou S, Diaz LA, Kinzler KW (2013). Cancer genome landscapes. Science.

[28] Pyne NJ, Pyne S (2010). Sphingosine 1-phosphate and cancer. Nature reviews.

[29] Spiegel S, Milstien S (2011). The outs and the ins of sphingosine-1-phosphate in immunity. Nat Rev Immunol.

[30] Lee H, Deng J, Kujawski M, Yang C, Liu Y, Herrmann A, Kortylewski M, Horne D, Somlo G, Forman S, Jove R, Yu H (2010). STAT3-induced S1PR1 expression is crucial for persistent STAT3 activation in tumors. Nat Med.

[31] Chen J, Tang H, Sysol JR, Moreno-Vinasco L, Shioura KM, Chen T, Gorshkova I, Wang L, Huang LS, Usatyuk PV, Sammani S, Zhou G, Raj JU, Garcia JG, Berdyshev E, Yuan JX (2014). The sphingosine kinase 1/sphingosine-1-phosphate pathway in pulmonary arterial hypertension. American journal of respiratory and critical care medicine.

