# Supplementary material for: Tumor suppressor PRSS8 targets Sphk1/S1P/Stat3/Akt signaling in colorectal cancer
Source: Oncotarget. 2016 Mar 31;7(18):26780–92. doi: 10.18632/oncotarget.8511 (PMC5042014; doi:10.18632/oncotarget.8511)
Supplement: Supplementary file 1 [file oncotarget-07-26780-s001.pdf]

Tumor suppressor PRSS8 targets Sphk1/S1P/Stat3/Akt signaling in colorectal cancer

SUPPLEMENTARY TABLE

Supplementary Table S1: Primers for qRT-PCR analysis and small interfering RNA sequences

Primers for qRT-PCR analysis

Gene name	Forward primer sequence	Reverse primer sequence
PRSS8	AGAGGACATGGTGTGTGCTG	GAGGCTGGAGTTCTGTCACC
GAPDH	GTCAAGGCTGAGAACGGGAA	AAATGAGCCCCAGCCTTCTC

Small interfering RNA sequences

Gene name-position	Sense (5'-3')	Antisense (5'-3')
PRSS8-1906	CAGGGCAGAAAUGAUUAAATT	UUUAAUCAUUUCUGCCCUGTT
PRSS8-932	GUAACUGCCUGUACAACAUTT	AUGUUGUACAGGCAGUUACTT
Sphk1-562	GAGGCUGAAAUCUCCUUCATT	UGAAGGAGAUUUCAGCCUCTT
Sphk1-1280	GCGUCAUGCAUCUGUUCUATT	UAGAACAGAUGCAUGACGCTT
Sphk1-1358	GCAGGCAUAUGGAGUAUGATT	UCAUACUCCAUAUGCCUGCTT
scrambled siRNA	UUCUCCGAACGUGUCACGUTT	ACGUGACACGUUCGGAGAATT
